# The Associations Between Physical Performance and Anthropometric Characteristics in Obese and Non-obese Schoolchild Handball Players

**DOI:** 10.3389/fphys.2020.580991

**Published:** 2021-01-22

**Authors:** Souhail Hermassi, Roland van den Tillaar, Nicola Luigi Bragazzi, René Schwesig

**Affiliations:** ^1^Sport Science Program, College of Arts and Sciences, Qatar University, Doha, Qatar; ^2^Department of Sport Sciences and Physical Education, Nord University, Levanger, Norway; ^3^Department of Health Sciences (DISSAL), Postgraduate School of Public Health, Genoa, Italy; ^4^Department of Orthopaedic and Trauma Surgery, Martin-Luther-University Halle-Wittenberg, Halle (Salle), Germany

**Keywords:** physical performance, youth players, anthropometrics, body fat, handball, body mass

## Abstract

This study investigated the relationship of body fat and fitness measures in schoolchild handball players. Twenty-eight young male handball players from handball first youth league volunteered for the present investigation (age: 10.9 ± 0.72 years; body mass: 54.8 ± 22.9 kg; height: 1.48 ± 0.10 m; body fat: 27.6 ± 9.23%). Measures included the Yo-Yo Intermittent Recovery Test level 1 (Yo-Yo IR1), jumping ability [squat and counter-movement jumps (SJ, CMJ)], and sprint tests (10 m, 15 m). Anthropometry was assessed by body mass, body mass index (BMI), and fat percentage (%BF). The power of the upper limb was measured as the total distance thrown overhead using a 2 kg medicine ball. Intrarater reliability for all parameters showed a coefficient of variation (CV) below 10% and an intraclass correlation coefficient (ICC) above 0.75. All ICC were excellent (ICC ≥ 0.96). Reliability as shown by the CV differed between 1.0 (sprint 15 m) and 5.6 (sprint 10 m). With the exception of medicine ball throw, we found significant differences between non-obese and obese in all performance parameters. The differences ranged from η*_p_*^2^ = 0.47 (sprint 10 m) to η*_p_*^2^ = 0.09 (medicine ball throw). The two-step-linear regression analysis using the predictors body height and body weight (step 1) and body fat (step 2) showed a marked increase of explained variance by adding body fat. The largest r^2^ changes were calculated for sprint 10 m (0.54), CMJ (0.49), and sprint 15 m (0.42). The lowest influence of the predictors was observed for medicine ball throw (step 1: *r*^2^ = 0.03, step 2: *r*^2^ = 0.07). With the exception of sprint parameters (β-coefficient sprint 10 m: -0.74; β-coefficient sprint 20: -0.66), a decrease of %BF leads to a higher performance in all parameters. %BF in youth handball players should be an important concern for practitioners working in this team sport in contrast to the frequently used BMI. It seems sensible and appropriate to engage very young children in physical activities such as team handball in order to improve their physical fitness. Decrease in% body fat could be considered both as a training and nutritional target to enhance and optimize sport performance-related outcomes.

## Introduction

Obesity in children represents a major public health concern (Boreham et al., 2004), as this epidemic has been linked to many diseases, in particular to chronic-degenerative disorders (Guo et al., 2002; Boreham et al., 2004). While affecting all age groups, overweight is a public health issue especially among subjects aged less than 15 years (Güngör, 2014; Hamano et al., 2015). While sport and physical activity are an effective tool for preventing the insurgence of obesity or at least counteracting and mitigating its burden, there exists a body of research with contrasting findings (Nelson et al., 2011). The various health and societal implications of excess body weight such as overweight and obesity are well established (Veugelers and Fitzgerald, 2005). Childhood overweight dramatically impact self-esteem, impairing cognitive and social development (Tremblay et al., 2000; Hesketh et al., 2004). Since childhood overweight often tends to persist into adulthood, an increasing number of adults is expected to be at higher risk of suffering from these conditions as well as from chronic-degenerative disorders, including cardiovascular disease, osteoarthritis, and malignancies (Dietz, 2004). Moreover, according to a number of investigations carried out in athletes aged from 11 to 19 years old, obesity was reported in 13% of the participants according to BMI vs. 6% based on percentage of body fat (Etchison et al., 2011). Hence, 62% classified as obese based on BMI were false positives (Etchison et al., 2011). In another study, 10% of boys playing rugby were classified as overweight according to percent body fat percentiles (Walsh et al., 2011). These investigations show a marked portion of youth athletes being still categorized as overweight or obese utilizing excess adiposity as a classification marker.

Even if handball is a rather popular sports discipline in European countries (Moreno et al., 2004), no investigation has ever been carried out to explore the prevalence rate of excess of body weight (namely, overweight, and obesity) in youth male handball players and their implications in terms of physical fitness in a sample of young Qatari handball players.

However, handball is an intermittent sports discipline where anthropometric and physiological parameters are crucial, including the skill to perform repeated explosive muscular contractions not only in adults but also in young players (Schwesig et al., 2017; Hermassi et al., 2019a,b). Athletes undergo a specific, long-term training in order to adapt their fitness, body composition, and anthropometric characteristics to this sport (Schwesig et al., 2017; Hermassi et al., 2019a,b). Chelly et al. (2015) have reported that the anthropometric features of the handball players have a major effect in terms of competitiveness. Moreover, the anthropometric profiling of players can be a promising strategy when discovering talent, enabling to identify both strengths and weaknesses, and to personalize the implementation of strength, and conditioning interventions (Fieseler et al., 2017). Many studies carried out among handball players have managed to detect statistically significant findings in terms of playing level and category for several physical, physiological, and anthropometric features (Šibila and Pori, 2009; Zapartidis et al., 2011; Karcher and Buchheit, 2014).

The impact of fitness, body composition, other relevant anthropometric variables and their associations with the specific discipline and the playing position have been described and reported for several sports (Mitchell et al., 2016; Lesinski et al., 2017; Fields et al., 2018). For instance, in a study investigating differences in terms of physical/anthropometric characteristics between male university students not engaged in sport and physical activity, and those engaged in various disciplines, such as athletics, rugby, soccer, competitive swimming, handball, and gymnastics, anthropometric characteristics and body composition were found to differ depending on the discipline (Tanaka et al., 1997). While a number of studies have contributed to a better understanding of the associations between isokinetic strength and sports performance-related outcomes in athletics and soccer (Watanabe et al., 2003; Miyamori et al., 2008), there is a dearth of data and information concerning differences in anthropometric characteristics and fitness between young handball players.

When devising and implementing *ad hoc* training interventions and identifying talented athletes among youth handball players, it is of paramount importance to assess their fitness, body composition, and other relevant anthropometric characteristics. Even though the previously mentioned researches have refined and improved our understanding of the complex interplay between anthropometric characteristics and physical fitness, there is a lack of data concerning this association in the specific sports discipline of handball. These data could have practical implications for sports coaches, scientists, and managers dealing with handball players, specifically with youth. Some studies have indeed shown how aerobic capacity and sprint and other physical fitness related components can be used to categorize handball players based on their different competitive levels (Šibila and Pori, 2009; Zapartidis et al., 2011; Karcher and Buchheit, 2014).

Moreover, the relationship between anthropometric features (such as body height, mass, and %BF), and physical fitness should be clarified in young handball players, since it impacts reflecting performance-related variables. As a consequence, long-term athletic development (LTAD) seems to be a particularly advantageous pathway in terms of the enhancement of the general health status, physical fitness, and youth sports participation (Cöte et al., 2007; Lloyd et al., 2015). Enhancing the physical abilities of children to optimize athletic success at an adult age is a major strategy, as outlined by earlier youth-based training programs (Bompa, 2000). In fact, LTAD model (Lloyd et al., 2015) incorporates the maturational status of the subject during childhood and offers unique opportunities for the young athletic development in terms of increased performance outcomes (Cöte et al., 2007). Providing young athletes of all age groups and skills with strategic programs aimed at fully developing their health level as well as their physical fitness is also of paramount importance to significantly increase participation rates in physical activities, curb the risk of sport- and activity-related injury, and to guarantee long-term well-being and health levels (Lloyd et al., 2015).

Furthermore, there is a significant lack of data specifically related to countries from the Gulf region, considering the unique features of this geographical area implying that research outcomes from other parts of the world cannot be generalized and directly translated/applied to this territory. Due to overcoming unhealthy habits, including sedentary lifestyles, the proportion of overweight children in Qatar (Al-Thani et al., 2018; Hermassi et al., 2020) is daily on the rise together with poor physical activity levels and unsatisfactory motor skills.

As such, the aim of this study was to assess the association between body composition-related variables (such as body height, body mass, and %BF), and physical fitness-related components measured in a sample of youth handball players, reflecting performance outcomes. We formulated the following working hypotheses: (1) %BF would negatively correlate with physical performance, even after taking into account other fundamental body composition-related variable measures such as body height and mass, (2) %BF is expected to highly contribute to performances requiring weight-bearing exercises, and (3) obese players can be expected to have lower fitness levels with respect to non-obese individuals with low fitness levels.

## Materials and Methods

### Participants

The present study was carried out in-season period, in the period ranging January–February 2020. Twenty-eight male handball players from the Qatar handball first league were recruited (age: 10.9 ± 0.72 years; body mass: 54.8 ± 22.9 kg; height: 1.48 ± 0.10 m; body fat: 27.6 ± 9.23%). They did not suffer from any musculoskeletal injuries in the 4 weeks before the beginning of the current investigation. Participants reported an average training of 4.2 ± 0.05 h/week, had an experience of at least 4 years, and took routine part in one match per weekend. The training comprised motor skills (60% of session time) and basic team handball techniques through playing games (40% of session time). In addition, players took part in weekly school physical education sessions, lasting for 40 min and consisting mainly of ball games. They did not train between the *pre-test* and 24 h after carrying out the test procedures. Exclusion criteria included suffering from any chronic-degenerative disease or orthopedic condition that could interfere with the participation in the training program, matches, or experimental tests. Before the start of the current investigation, all participants and parents were thoroughly advised about the potential risks and benefits associated with the participation in this study. All players and/or their parents/guardians provided a signed, written, informed consent. Players were thoroughly familiarized with all the study procedures and were advised about the possibility of withdrawing their consent to participate in the study at any time without experiencing any penalty. This study protocol was fully reviewed and received approval by the national university institutional review board (Approval Number: QU-IRB 1163-EA/19) for human subjects and is compliant with the requirements of the Declaration of Helsinki.

### Procedures and Evaluations

Test measurements were carried out on a regular indoor handball court, at the same time of day (from 5:00 to 7:00 p.m.) and under similar experimental and environmental conditions (temperature 20.5 ± 0.5°C, relative humidity 60 ± 5%), at least 3 days after a competition. Participants were thoroughly instructed to preserve their usual dietary habits, while abstaining from consuming caffeine-containing beverages or food for 4 and 2 h prior to testing, respectively. They were also asked not to conduct any vigorous physical activity for 24 h prior to testing. The investigation comprised separate test blocks to measure general and team handball specific physical performance characteristics (Wagner et al., 2018). The rationale for the test selection was the relevance for handball and the evaluation of the following dimensions:

1. Sprinting performance (10 m, 15 m),
2. Jumping performance (CMJ, SJ),
3. Throwing performance (medicine ball overhead throw),
4. Endurance performance (Yo-Yo IR1).

The tests were conducted over a period of 4 days in a fixed order to elicit similar fatigue effects between players. On the first day, anthropometric features were measured, followed by vertical jump tests (SJ and CMJ). The second day was dedicated to sprint performance. On the third day, the agility T-Half test and medicine ball throw were evaluated, followed by the Yo-Yo IR1 on the fourth day.

Two weeks after the initial testing period, the physical fitness tests conducted from days 1 to 3 (i.e., SJ, CMJ, sprints, T-Half agility, medicine ball throw) were repeated to allow the assessment of their test–retest reliability. Only the scores of the second set of tests were analyzed. Anthropometric features measurements and the Yo-Yo IR1 test were only conducted once during the initial testing period due to reasons concerning the organization of the study (i.e., time and players’ schedules).

### Day 1

#### Anthropometry

Measured anthropometric characteristics included standing height (Holtain stadiometer, Crosswell, Crymych, Pembrokeshire, United Kingdom, accuracy of 1 mm) and body mass (model TBF 105; Tanita Corporation of America, Inc., Arlington Heights, Illinois) and were recorded to the nearest 0.1 cm and 0.1 kg, respectively. Body mass index (BMI) was computed as the ratio between body mass (measured in kg) and body height squared (measured in m^2^). Skinfold thickness was calculated to the nearest mm, except for low values (usually 5 mm or less) when it was computed to the nearest 0.5 mm. These readings were performed at four sites on all individuals, at the biceps, triceps, subscapular, and supra-iliac areas. These were usually conducted on the right side of the body with the participants standing up and being relaxed, even though we could not find any statistically significant difference between measurements on either side of the body (Womersley and Durnin, 1973). Skinfold thickness was with a Harpenden caliper (Baty International, Burgess Hill, Sussex, United Kingdom). Readings were taken in duplicate at each site, and the average of the two measurements was recorded. If the two readings varied by more than 2 mm, a third one was performed, and the closest two measurements were averaged. The four skinfold measurements were summed, in order to estimate body fat according to the sex- and age-specific equation (Womersley and Durnin, 1973) as previously reported in young athletes (Chelly et al., 2015). However, the mean percentage of body fat was computed from the biceps, triceps, subscapular, and suprailiac skinfolds, utilizing the following equation (Womersley and Durnin, 1973):

(1) %Bodyfat=[4.95/(Density-4.5)]•100

(2) WhereDensity=1.162--0.063(LOGsumof4skinfolds).

Participants were then assigned to one of two %BF groups (i.e., obese or non-obese) based on age-stratified norms for males (Lohman et al., 1997) on %BF into normal fat (i.e., non-obese) (< 17 and < 25%) and over-fat (i.e., obese) (≥ 25 and ≥ 31% body fat) categories for males on age-stratified norms.

#### Squat (SJ) and Counter Movement Jump (CMJ) Tests

Before the jumping tests, the players were engaged in a similar general warm-up procedure that comprised 5 min of running, stretching of lower limbs muscles and 2 min of jumping exercises. SJ and CMJ heights were measured using the Optojump photoelectronic cells (Optojump Next, Microgate, Italy) (Glatthorn et al., 2011). Jump heights were assessed from the recorded contact and flight time of vertical jumps with an accuracy of 1/1,000 s (1 kHz). The SJ started at a 90° knee angle; without performing any downward movement, players carried out a vertical jump by pushing upwards with their legs. The CMJ started from an upright position, with players performing a rapid downward movement to a knee angle of approximately 90°, arms akimbo and simultaneously beginning to push-off, after being thoroughly advised to jump as fast and high as possible. Both tests were conducted without an arm swing by keeping the hands fixed at the level of the pelvis and with knees and ankles extended at take-off and landing. The largest of four jumps was recorded for each test, and a 30 s recovery was granted between each jump.

### Day 2

#### Sprint Tests

Before the sprint testing, each participant performed a 5 min warm up, comprising 3 min of running, change in direction activities, and dynamic stretching. Participants ran 15 m from a standing position, with the front foot 0.2 m behind the starting photocell beam. Times at 15 and 30 m were measured by utilizing paired photocells (Racetime 2 SF, Microgate, Italy) that were located 1 m above the ground at the start and finish lines. Three trials were separated by 6–8 min of recovery, and the fastest trial was retained for further analyses.

### Day 3

#### Ability to Change Direction (T-Half Tests)

**FIGURE 1:**
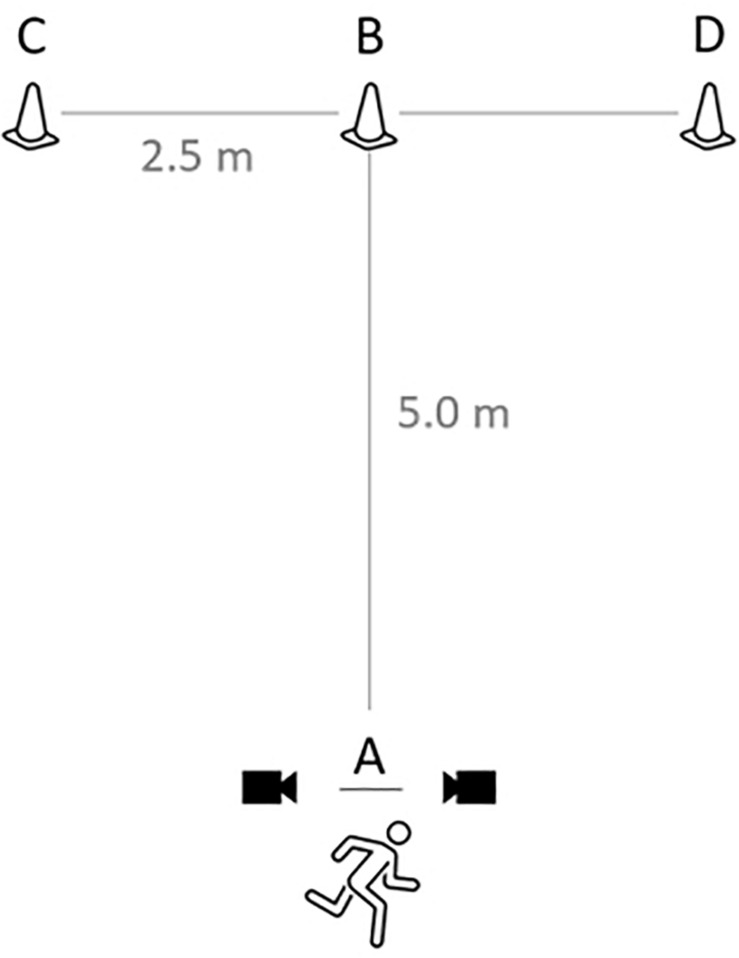
Schematic presentation of the t-half test modified by Sassi et al. (2009).

A 10 min warm-up consisting of jogging, lateral displacements, dynamic stretching and jumping was conducted before the beginning of the tests. T-Half tests (Sassi et al., 2009) data were recorded using electronic timing sensors (photocells, Kit Racetime 2 SF, Microgate, Italy) set at 0.75 m above the floor, 3 m apart and facing each other at the starting line A (Figure 1). Testing began with the front foot placed 0.2 m behind line A. Discretionally, players participants sprinted forward to cone B and touched its base with their right hands. Facing forward and without crossing feet, they shuffled to the left to cone C and touched its base with their left hands. They then shuffled to the right to cone D and touched its base with their right hands, subsequently running back to the left to cone B and touching its base. Finally, they ran backward as quickly as possible, returning to line A. Anyone who crossed one foot in front of the other, failed to touch the base of a cone, and/or failed to face forward throughout had to repeat the test. Subjects repeated the test until two successful trials were done, with 3 min of rest given between each trial, and only the best trial was considered in the analyses.

#### Medicine Ball Overhead Throw

Before the medicine ball overhead throw testing, each participant carried out a 5 min warm up, which comprised 3 min of running and dynamic activities. Medicine ball throws were conducted using 21.5 cm diameter 3 kg rubber medicine balls (Tigar, Pirot, Serbia). All individuals were first familiarized during *ad hoc* preliminary sessions. A brief overview of the optimal technique to utilize was provided, suggesting a release angle in order to attain a maximum distance of throw (Negrete et al., 2010). The sitting participant had to grasp the medicine ball with both hands, and on the given signal forcefully had to push the ball from the chest. The score was calculated from the front of the sitting line to the place where the ball landed. Three trials were carried out and interspersed with 1 min of rest. The criteria for considering a test measurement valid and acceptable were recording of the better of two definitive trials. The distance of the throw was measured in 0.01 m.

### Day 4

#### The Yo-Yo Intermittent Recovery Test Level 1 (Yo-Yo IR1)

The Yo-Yo IR1 was carried out following the instructions provided by Krustrup et al. (2003). The reliability of the test has been measured yielding a coefficient of variation of 3.6% and an intraclass correlation (ICC) coefficient of 0.94 (Krustrup et al., 2003). A standardized warm up consisted of 5 min of low-intensity running. Players conducted 20 min shuttle runs at increasing velocities until exhaustion, with 10 s intervals of active recovery (2 × 5 m of jogging) being granted between runs. The test was terminated if players failed twice to reach the front line in time (objective criterion) and/or felt unable to complete another shuttle at the requested speed (subjective criterion). The total distance covered was retained as the test score and then analyzed.

### Statistical Analyses

All studied variables were expressed performing descriptive statistics as means, standard deviations (SDs), with their minimum and maximum values (range), and their 95% confidence intervals (95% CI). Mean differences between non-obese and obese were tested using one-factor univariate general linear model (Bortz, 1999). Differences between means were considered statistically significant if *p*-values were less than 0.05 and partial eta-squared (η*_p_*^2^) values were higher than 0.15 (Richardson, 2011). A power calculation (nQuery Advisor 4.0; Statistical Solutions, Saugus, MA) showed that with a sample size of seven participants in each group, the study had 80% power to detect a difference between means of 3.0 cm (main outcome: CMJ height) using a two-sided *t*-test with a significance level of *p* < 0.05, under the assumption of a pooled standard deviation of 2.0 cm (Bortz, 1999).

A two-step-linear regression analysis (with “inclusion” as method) were conducted to shed light on the relationships between anthropometric parameters (%BF, body weight, body height) and performance parameters (e.g., SJ, CMJ, sprint 10 m). In the first step, body weight and body height were used as predictors with the regression model. In order to judge the influence of body fat and the additional explained variance, in the second step, body fat was included in the regression analysis as third predictor. We reported r^2^ and the change of r^2^ between steps 1 and 2, and the standardized regression coefficient ß for all predictors and steps. The magnitude of correlations (r) between measures was interpreted according to the following rule of thumb: if r was < 0.1, the correlation was deemed trivial; in the range 0.1–0.3, it was judged small; in the range 0.3–0.5, moderate; in the range 0.5–0.7, large; in the range 0.7–0.9, very large;, and in the range 0.9–1.0, almost perfect (Cohen, 1988). Therefore, *r*^2^ > 0.5 (explained variance > 50%) was defined as relevant and marked in bold (Table 3).

Intraclass correlation coefficient (ICC) and the coefficient of variation (CV) were calculated in order to quantitatively assess the intrarater reliability (Schrama et al., 2014). Interpretation of reliability values was done based on several rules of thumb elaborated by Shrout and Fleiss (1979); Hopkins (2000), and Portney and Watkins (2009). The ICC displayed an excellent relative reliability if the value was greater than 0.75, a fair-to good reliability in the range 0.40–0.75, and a poor reliability if lower than 0.40 (Cohen, 1988). ICC values may reflect inter-subject variability of scores because a large ICC may be reported despite poor trial-to-trial consistency if the inter-subject variability is rather high (Weir, 2005; Portney and Watkins, 2009). The CV, which can be considered as an indicator of measurement variability and random error, was computed after log-transforming the obtained raw data (Hopker et al., 2010). The CV can be defined as a reliability measure with values lower than 10% being usually employed as a threshold to differentiate between good and poor reliability (Cormack et al., 2008; Brughelli and Van Leemputte, 2013).

All statistical analyses were performed using SPSS version 25.0 for Windows (SPSS Inc., Chicago, IL, United States).

## Results

### Intrarater Reliability

All five performance-related variables exhibited an excellent relative reliability (ICC ≥ 0.75). The ICC varied from 0.96 (CMJ, sprint 10 m) to 1.00 (sprint 15 m). All variables under study also displayed an excellent absolute reliability, with a CV below 10% (Table 1). Only the 10 m sprint times was characterized by a weaker absolute reliability (CV = 5.6%, CI: 7.1–13.6), specifically when compared with the other parameters (range: 1.0–2.8%).

**TABLE 1:** Comparison of test measurements obtained from the two testing sessions prior to the intervention (*n* = 28).

test	Session one mean ± SD	Session two mean ± SD	ICC (95% CI)	CV (%) (95% CI)
Squat jump (cm)	18.1 ± 4.39	17.2 ± 4.41	**0.99** (0.45–1.00)	**2.1** (1.6–3.1)
Countermovement jump (cm)	22.2 ± 2.86	21.1 ± 2.85	**0.96** (0.00–0.99)	**1.7** (1.3–2.6)
Sprint 10 m (s)	2.48 ± 0.52	2.50 ± 0.47	**0.96** (0.91–0.98)	**5.6** (4.3–8.7)
Sprint 15 m (s)	3.54 ± 0.52	3.58 ± 0.51	**1.00** (0.99–1.00)	**1.0** (0.7–1.4)
Medicine ball overhead throw (m)	4.88 ± 0.90	4.74 ± 0.86	**0.98** (0.91–1.00)	**2.8** (2.1–4.2)
Biceps skinfold (mm)	13.1 ± 6.76	13.2 ± 6.77	**1.00** (1.00–1.00)	**3.1** (2.4–4.7)
Triceps skinfold (mm)	20.4 ± 10.4	20.2 ± 10.0	**1.00** (1.00–1.00)	**4.8** (3.7–7.4)
suprailiac skinfold (mm)	21.0 ± 11.7	21.0 ± 11.8	**1.00** (1.00–1.00)	**1.9** (1.4–2.8)
Subscapular skinfold (mm)	20.0 ± 12.0	19.9 ± 11.9	**1.00** (1.00–1.00)	**2.0** (1.5–3.0)

*Descriptive statistics (mean ± SD) and intrarater reliability analysis calculated for each test are presented. ICC ≥ 0.75 and CV ≤ 10% are highlighted in bold.*

The relative reliability regarding the anthropometric parameters (Table 1) was on the same highest level for all parameters (ICC = 1.00). The CV was markedly below 10% and ranged from 1.9 (Suprailiac skinfold) to 4.8% (Triceps skinfold).

### Body Fat Distribution of the Participants and Comparison of Non-obese vs. Obese

Almost two thirds of the subjects (19/28, 64%) were obese (Table 2). The body fat values ranged from 12.6 to 44.9%. Three children (11%) showed a body fat above 40%.

**TABLE 2:** Participants’ physical characteristics by category of percent body fat (non-obese and obese) and comparison of parameters between the non-obese and obese group based on the 1st testing set.

	Non-obese (*n* = 10)	Obese (*n* = 18)	Total (*n* = 28)	ANOVA	
	
				*p*	η*_p_*^2^
Age (years)	11.2 ± 0.79	10.8 ± 0.65	10.9 ± 0.72	0.138	0.083
**Anthropometric parameters**					
Body height (m)	1.50 ± 0.13	1.47 ± 0.08	1.48 ± 0.10	0.557	0.013
Body mass (kg)	55.5 ± 21.9	54.4 ± 24.0	54.8 ± 22.9	0.908	0.001
BMI (kg⋅m^–2^)	24.2 ± 7.04	24.4 ± 8.70	24.4 ± 8.01	0.932	0.000
Body fat (%)	17.6 ± 4.03	33.1 ± 5.96	27.6 ± 9.23	**< 0.001**	**0.674**
**Performance parameters**					
CMJ (cm)	24.1 ± 2.23	21.2 ± 2.67	22.2 ± 2.86	**0.007**	**0.247**
SJ (cm)	20.7 ± 4.01	16.7 ± 4.04	18.1 ± 4.39	**0.020**	**0.191**
Sprint 10 m (s)	2.95 ± 0.45	2.21 ± 0.35	2.48 ± 0.52	**< 0.001**	**0.468**
Sprint 15 m (s)	3.94 ± 0.47	3.32 ± 0.41	3.54 ± 0.52	**0.001**	**0.333**
Yo-Yo IR1 distance (m)	784 ± 366	462 ± 316	577 ± 364	**0.022**	**0.186**
Medicine ball overhead throw (m)	5.23 ± 1.32	4.69 ± 0.50	4.88 ± 0.90	0.129	0.086

*Data are presented as means ± standard deviation. Significant differences (p < 0.05 and η_p_^2^ > 0.15) marked in bold.*

**TABLE 3:** Summary of the linear regression analyses for anthropometric variables predicting performance parameters.

				Standardized regression coefficient β		
				
		*R*^2^	Change	Body height (cm)	Body mass (kg)	Body fat (%)
Sprint 10 m (s)	Step 1	0.04		−0.11	0.27	
	Step 2	**0.58**	0.54	0.06	0.16	−0.74
Sprint 15 m (s)	Step 1	0.03		0.04	0.13	
	Step 2	0.45	0.42	0.20	0.03	−0.66
CMJ (cm)	Step 1	0.14		−0.44	0.12	
	Step 2	**0.63**	0.49	−0.28	0.01	−0.71
SJ (cm)	Step 1	0.07		−0.36	0.25	
	Step 2	0.33	0.26	−0.24	0.17	−0.51
Medicine ball overhead throw (m)	Step 1	0.03		0.13	0.05	
	Step 2	0.07	0.04	0.18	0.02	−0.21
Yo-Yo IR1 distance (m)	Step 1	0.14		−0.50	0.36	
	Step 2	0.47	0.33	−0.37	0.27	−0.58

*Body height and body mass were entered as predictors in step 1, while percent body fat (%) was added in step 2. Standardized regression coefficient (β) are given for each predictor. R^2^ > 0.5 marked in bold.*

Both groups showed no significant differences regarding anthropometric parameters. Of course, the distinguishing criterion body fat was significantly different between both groups (17.6 ± 4.03 vs. 33.1 ± 5.96%; η*_p_*^2^ = 0.674).

**FIGURE 2:**
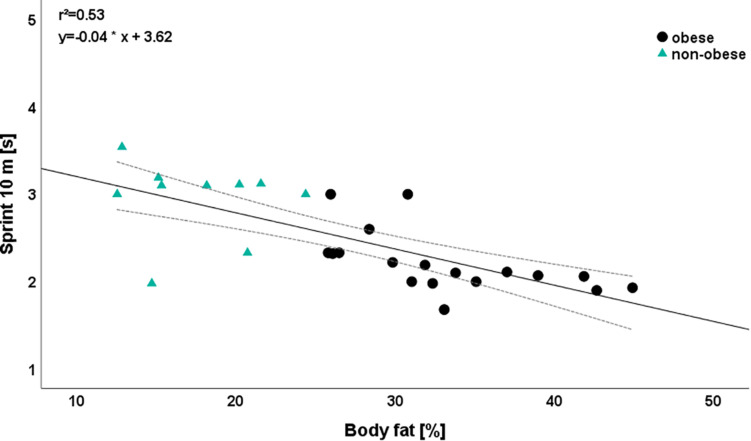
Relationship between body fat and sprint 10 m depending on group. Please note that one dot can represent several subjects.

In contrast and apart from medicine ball throw, significant mean differences were found for all performance parameters. The largest differences were calculated for sprint 10 m (η*_p_*^2^ = 0.468) and sprint 15 m (η*_p_*^2^ = 0.333), whereas the smallest difference was observed for medicine ball throw (η*_p_*^2^ = 0.086). In both sprint parameters, the obese group showed a higher performance level than the non-obese subjects (Figure 2).

### Relationships Between Anthropometric and Physical Performance Parameters

Before the two-step-linear regression analysis, the collinearity of anthropometric data (body height, body weight, body fat) was checked by using the bivariate Pearson’s product moment correlation (r) calculation:

• Body weight/body height: *r* = 0.670,
• Body weight/body fat: *r* = 0.008,
• Body height/body fat: *r* = 0.134.

The two-step-linear regression analysis with the predictors body height and body weight (step 1) and body fat (step 2) displayed a marked increase in explained variance by adding body fat in the second step (Table 3). The r^2^ changes between the first and second step ranged from 0.04 (medicine ball overhead throw) to 0.54 (sprint 10 m).

**FIGURE 3:**
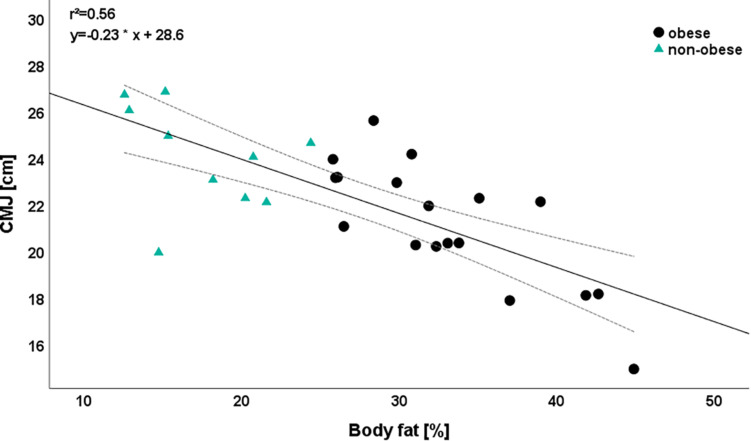
Relationship between body fat and counter-movement jump (CMJ) depending on the group. Please note that one dot can represent several subjects.

The largest additional explained variances were calculated for sprint 10 m (0.54), CMJ (0.49), and sprint 15 m (0.42). Regarding medicine ball overhead throw, we observed no relevant influence of any predictor (step 1: *r*^2^ = 0.03, step 2: *r*^2^ = 0.07). With the exception of sprint parameters (β-coefficient sprint 10 m: −0.74; β-coefficient sprint 20: -0.66), a decrease in body fat leads to a higher performance in all parameters. For example, a reduction of 1% body fat induced an increase in CMJ jumping height from 0.71 cm (Figure 3).

On the other hand, the same reduction in body fat was related with a decrease in sprint performance (sprint 10 m: 0.74 s; sprint 15 m: 0.66 s). This result corresponds to the results of variance analysis (Table 2), according to the obese subjects (e.g., sprint 10 m: 2.21 ± 0.35 s), which are significantly (e.g., sprint 10 m: *p* < 0.001, η_p_^2^ = 0.468) more powerful in sprinting parameters than non-obese children (e.g., sprint 10 m: 2.95 ± 0.45 s).

## Discussion

The main objective of the present investigation was to explore how %BF can contribute to identifying the determinants of the variability in basic field physical fitness related measures in schoolchild handball players. Major results provided support to previously published findings concerning the negative impact of high %BF on both aerobic and anaerobic fitness related components, thereby providing support to our first working hypothesis. After taking into account both body height and mass, %BF still explained a relevant proportion of specific performance related variables. In fact, body height and mass seemed to play only a small role in CMJ and aerobic capacity testing, whereas for instance, considering sprint tests (like running tests) a higher %BF seems to be advantageous in terms of sprinting performance. The need for a differentiated view of %BF depending on performance parameter is a very important finding of our investigation. The latter only partly confirms our second working hypothesis which assumed a significant relationship between %BF and scores achieved on running tests (such as weight-bearing). The current investigation succeeded to identify the contribution of %BF to performance related outcomes in a sample of youth handball players stratified according to their body height and body mass. Improving %BF should be considered as a well-designed exercise program and nutrition goal to enhance both the aerobic capacity and the anaerobic components such as agility, sprinting, throwing, and jumping. Obviously, in this age, the importance of %BF regarding physical performance is much higher than from the body height and body mass.

### Anthropometric and Aerobic Capacity

Players were categorized based on age-stratified cut-off values of %BF in obese or non-obese. Their BMI values did not vary, while statistically significant differences could be detected in %BF and all skinfold measurements. Such a finding demonstrates that BMI is a poor indicator for discriminating between obese and not-obese athletes (Kruschitz et al., 2013). However, obesity seemed to impact the aerobic performance to a much larger extent, with a statistically significant (*p* = 0.022) 19% difference (η*_p_*^2^ = 0.186, Table 2) being detected between groups. This result is in line with previously published studies indicating that having more %BF is associated with lower aerobic fitness in children and adolescents (Liao et al., 2013; Georgeson et al., 2017). This correlation has been relatively well explored in the general youth population (Burns et al., 2013; Georgeson et al., 2017).

The relationships between cardio-respiratory fitness and health level, especially cardiovascular health, are well-known in the existing scholarly literature, and body composition has shown correlation with health status in both children and adults (Alricsson et al., 2008; Nikolaidis, 2012a,b). Previously published studies have demonstrated an association between fat excess and lower cardiovascular fitness and power/strength as well (Burns et al., 2013; Liao et al., 2013). Other studies (Ekelund et al., 2007) have shown a strong negative association between body fat content and fitness levels in pre-pubescents aged from 7 to 12 years old.

Our findings partially support previous studies investigating general (Bovet et al., 2007; Artero et al., 2010) and sport populations (Chen et al., 2006). Nikolaidis (2012b) demonstrated that normal-weight male children and adolescents aged 6–18 years old overperformed in cardio-respiratory endurance, in muscle strength and endurance with respect to their overweight peers. Generally speaking, investigations have found that an increase in fat or decrease in muscle mass results into greater functional disability and lower physical performance, while muscle and fat mass have been considered as key factors related to a declining physical functioning. On the other hand, subjects with high body fat exhibited lower values of VO_2max_ in a statistically significant fashion (Nikolaidis, 2012a). In line with the aforementioned study, Mak et al. (2010) also revealed higher values in cardio-respiratory endurance and muscle endurance in individuals with normal-weight compared to those overweight in male adolescents 12–18 years. In a corresponding age group of male adolescents (aged from 13 to 18.5 years old), Artero et al. (2010) demonstrated that overweight participants reported lower aerobic performances in most of the tests assessed. The findings of this investigation were also in line with those reported by Bovet and colleagues (Bovet et al., 2010) who recruited a sample of male adolescents aged 12–15 years old and with those of Duvigneaud et al. (2008).

Due to the negative associations that %BF had with the cardio-respiratory fitness scores compared to BMI, these measures may be compared with BMI in the effort to identify children with less than adequate physical fitness.

### Anthropometric and Anaerobic Capacity

The previously mentioned findings on the groups’ comparative analyses were based on a dichotomic categorization related to pre-defined %BF cut-off values extracted from the existing scholarly literature (Lohman et al., 1997). Even though results are in line with the literature, it was necessary to consider the broader impact of %BF, as a continuous variable, on performance characteristics. Stepwise regressions exploring the contribution of %BF to essential performance features in handball shed light on the specific role of body fatness after adjusting for body height and mass that play a key role in any physical testing. We could not find any relevant association (step 1: ß < 0.30) between body height and body mass and sprinting performances (i.e., 10 and 15 m sprints) and medicine ball throw. Furthermore and based on the first step, jumping performances and Yo-Yo IR1 were mostly explained by body height (step 1) than by body mass. Apart from the step within the regression model, the parameter medicine ball throws did not show any relationship to one of the three anthropometric variables. The latter seems to suggest that previously published reports showing statistically significant associations between BMI and physical fitness do not completely underpin the effects of body fatness separately, and interpretations concerning excess of weight such as overweight and obesity BMI-based classifications warrant caution. Body height was the strongest predictor of scores on the CMJ and Yo-Yo IR1 tests compared to body mass. The addition of %BF in step 2 showed a significant unique contribution to the latter performances, especially in sprinting and jumping performances. Interestingly, the %BF was the strongest predictor of all physical performance parameters, while body mass did not show a relevant predicting power.

In the existing scholarly literature, a huge body of research has consistently shown that overweight and obese youth tend to have a poorer performance in motor tests than their normal weight peers (Duvigneaud et al., 2008). A huge body of research focused on the youth proved that excess in body weight (overweight and obesity) negatively affects the level of basic motor skills and motor performance (Deforche et al., 2003; D’Hondt et al., 2013). In more detail, severe deficits in fundamental movement skills diagnosed as developmental coordination disorder were proved to be linked to high %BF (Faught et al., 2005; Cairney et al., 2012).

The most important finding was achieved from the comparison of the various %BF values, which revealed that the highest body fat quartile scored lower in most of the tests. This seems to suggest that a threshold does exist in %BF, over which physical fitness is affected largely. In fact, body correlates with functional limitations in muscle performance and increased probability of developing a functional disability such as strength, mobility, postural, and dynamic balance limitations (Mota et al., 2002). Maybe this is one of the reasons of the lack of association between medicine ball overhead throw and %BF.

A key result was obtained from comparing the two groups with different %BF, which showed that the highest %BF scored lower in most of the tests. This seems to suggest that a cut-off value might exist in body fat, above which physical fitness is largely affected. Furthermore, regression analyses indicated that regardless of set cut-off values, increased BF percentages would have a strong negative impact on performance parameters with the exception of sprinting performance, which are essential components of team handball. The sprinting performance is increasing (in the sense of time reduction) of 0.74 s (10 m) and 0.66 s (15 m), if the %BF is increasing by 1%. The relationship between body fat and sprint performance related to youth players who are advanced in age may be due to maturation, with large differences in anthropometrics due to various patterns in the growth spurt. The growth spurt is generally preceded by a quick increase in body fatness, a phenomenon known as the “pre-pubertal fat wave” (Tanner, 1989). However, a highly significant variability in timing biological maturation can be reported between individuals of the same sex resulting in early and late matures (Tanner, 1989; Armstrong and McManus, 2011) may imply a substantial difference in body mass and fat associated with sprint performance. Results suggest the importance of devising *ad hoc* programs targeting weight/fat control for optimizing and improving sports performance-related outcomes. Therefore, handball clubs and other sports organisms should have the responsibility of devising exercise-training and conditioning plans with the specific aim of modulating body mass and fat.

Young handball players from the Qatar handball first league exhibited a %BF value of 27.7, which is relatively high for youth athletes. An effort to shed light on the impact of excessive fatness on performance-related variables among the youth handball players is suggested in order to develop and implement *ad hoc* exercise plans aimed at enhancing body mass status.

### Limitations of the Study

The major shortcoming of the current investigation is its cross-sectional design that sexual maturation was not incorporated. An evaluation of %BF using DEXA could improve the interpretation of the results besides the skinfolds. In addition, the sum of more than four skinfolds could be considered in order to evaluate the %BF of the lower and upper body. Future high-quality studies should incorporate also sexual maturation when assessing the relationship between anthropometry and physical performance in youth handball players. A more thorough look at the fat distribution (e.g., from several skinfold measures) and its association to fitness-related variables would enable to better understand performance. Since the study design was cross-sectional, it was not possible to explore the causal nature of the studied relation. Moreover, further studies should incorporate laboratory tests of fitness, rather than field tests.

## Conclusion

Young handball players categorized as obese according to %BF exhibited lower scores in basic physical performance tests. In more detail, increased %BF values seemed to negatively impact running performance, vertical jump, and aerobic capacity after adjusting for body height and mass. We can speculate that both body mass status and the ratio between the amount of body fat and lean mass plays a major role in developing athletes. BMI measurement does not allow to properly discriminate between fat and lean masses, and as such is a poor predictor of physical performance-related variables in handball players. More focus should be given to multi-factorial variables to broaden the exploration of intra- and intercorrelation between physiological and anthropometric characteristics, like %BF and physical performance.

## Data Availability Statement

The raw data supporting the conclusions of this article will be made available by the authors, without undue reservation.

## Ethics Statement

The studies involving human participants were reviewed and approved by the Qatar university institutional review board (Approval Number: QU-IRB 1163-EA/19). Written informed consent to participate in this study was provided by the participants’ legal guardian/next of kin.

## Author Contributions

SH and RS: formal analysis, investigation, project administration, and supervision. SH, RS, and NB: methodology. SH and NB: writing—original draft. SH, RT, and NB: writing—review and editing. All authors contributed to the article and approved the submitted version.

## Conflict of Interest

The authors declare that the research was conducted in the absence of any commercial or financial relationships that could be construed as a potential conflict of interest.
